# Household Hints and Recipes

**Published:** 1875-10

**Authors:** 


### Household Hints & Recipes.

PRETTY RED DYE FOR WOOD.—To 2 lbs. genuine Brazil dust add 4 gallons water. Place the articles, immersed in this liquid, in a suitable vessel, boil them for three hours and let them cool, then add 2 oz. each of alum and aquafortis, and keep lukewarm until the required shade is obtained.

---

POTATOES SAUTEES.—These are even more agreeable with meat than fried potatoes. Cold boiled potatoes are sliced up, and tossed up in a saucepan with butter, mixed with a little chopped parsley, till they are lightly browned. Pure goose or other dripping is by many cooks preferred to butter for this purpose.

---

POTATOES SOUFFLEES.—These delicious blistered potatoes are prepared as follows: The potatoes, if small, are simply cut in halves; if large, cut into three or more slices; these are fried in the usual way, but are taken out before they are quite done, and set aside to get cold; when wanted they are fried a second time, but only until they are of a light golden color, not brown.

---

VIOLET POWDER.—The London *Chemist and Druggist* gives this recipe: Powdered starch or potato farina 28 lbs., orris powder 1 lb. This will require about an ounce of perfume, varying according to fancy. A mixture of ambergris and bergamot, with a little musk, is a favorite odor, and some makers add a few drops of oil of rhodium. The powder should be sifted.

---

ERASIVE SOAP, TO REMOVE GREASE AND STAINS FROM CLOTHING.—Two pounds of good Castile soap, half a pound of carbonate of potash, dissolved in half a pint of hot water. Cut the soap in thin slices; boil the soap with the potash until it is thick enough to mould in cakes; also add: alcohol half an ounce, camphor half an ounce, hartshorn half an ounce; color with half an ounce of pulverized charcoal.—*Scientific American.*

---

PUREE OF POTATOES.—This differs from mashed potatoes only in the employment of more milk and butter, and in the whole being carefully reduced to a perfectly smooth, thick, cream-like mixture. Where economy is a great object, and where rich dishes are not desired, the following is an admirable mode of mashing potatoes: Boil them till thoroughly done, having added a handful of salt to the water, then dry them well, and with two forks placed back to back, beat the whole up till no lumps are left. If done rapidly, potatoes thus cooked are extremely light and digestible.

---

A VERY HIGHLY ILLUMINATING OIL. — The *English Mechanic* gives this recipe: Pound in a mortar 2 oz. of camphor, slightly moistened with alcohol; add a pint of sperm oil, and stir till dissolved. Light the wick, after being thoroughly soaked, and let it burn for a few minutes; then blow out and re-trim with exactness, in order to secure an even, smokeless flame, which can be better done after the wick has been slightly charred.

---

HARMLESS COSMETIC POWDERS.—The apothecaries of Copenhagen have agreed on the substitution of certain harmless compounds for the numerous poisonous face powders now commonly used. In avoirdupois weight, the proportions of the ingredients will be about as follows: For white powder, oxide of zinc, 1 oz.; wheat starch, 9 oz.; oil of rose, three drops; for red powder, carmine, 1 oz.; carbonate of magnesia, 4 oz.

---

ADHESIVE LABELS.—Dissolve 1½ ozs. common glue, which has lain a day in cold water, with some candy sugar and ¾ oz. gum arabic, in 6 ozs. hot water, stirring constantly until the whole is homogeneous. If this paste is applied to labels with a brush and allowed to dry, they will then be ready for use on merely moistening with the tongue.

---

RHUBARB MARMALADE.—The following is an English recipe: Take 1¼ lbs. of rhubarb, 1 lb. of lump sugar, one lemon. Cut the rhubarb (after peeling) into square pieces, the shape and size of dice; chip the lemon on the top of it; cover the whole in an earthen vessel with fine white sugar, and let it stand at least 12 hours. Next morning, pour off the liquid (leaving the rhubarb behind,) and boil the juice for some time. The exact length of time cannot be fixed, as some kinds of rhubarb produce more liquid than others, but it must be boiled until it becomes syrupy. Next put the lemon into the syrup, also the rhubarb, and boil all together till the rhubarb becomes tender. Place in jars, and treat exactly as is done with preserves. If a ginger flavor is wished, put some of the best ginger (bruised) into the liquid when it is just poured off the rhubarb, and boil it along with it.

